# Peripapillary RNFL cross-sectional area and its association with other parameters in a Chinese population

**DOI:** 10.1186/s12886-024-03481-y

**Published:** 2024-06-17

**Authors:** Yan Yanni, Wang Qian, Wei Wenbin

**Affiliations:** Beijing Key Laboratory of Intraocular Tumor Diagnosis and Treatment, Beijing Ophthalmology & Visual Sciences Key Lab, Medical Artificial Intelligence Research and Verification Key Laboratory of the Ministry of Industry and Information Technology, Beijing Tongren Eye Center, Beijing Tongren Hospital, Capital Medical University, Beijing, 100730 China

**Keywords:** Peripapillary RNFL cross-sectional area, Ocular magnification, Optic disc area

## Abstract

**Background:**

Quantitative analysis of retinal nerve fibers is important for the diagnosis and treatment of optic nerve diseases. Peripapillary retinal nerve fiber layer (RNFL) cross-sectional area may give a more accurate quantitative assessment of retinal nerve fibers than RNFL thickness but there have been no previous reports of the peripapillary RNFL cross-sectional area or other parameters. The purpose of the current study was to determine peripapillary RNFL cross-sectional area and its association with other factors in an adult Chinese population.

**Methods:**

RNFL cross-sectional area was measured during peripapillary circular optical coherence tomography (OCT) scan with a diameter of 12° centered on the optic disc. Correlation between RNFL cross-sectional area and other parameters was evaluated by linear regression analysis in a cross-sectional study of an adult Chinese population.

**Results:**

A total of 2404 eyes from 2404 subjects were examined. Multivariate linear regression analysis showed that larger RNFL cross-sectional area correlated with younger age (*p* < 0.001), female gender (*p* = 0.001), no history of diabetes (*p* = 0.012) and larger optic disc area (*p* < 0.001).

**Conclusions:**

Peripapillary RNFL cross-sectional area is correlated positively with optic disc area, suggesting that eyes with larger optic discs have thicker RNFL. Further studies are needed to confirm whether this correlation is due to differences in the numbers of retinal nerve fibers or other factors.

## Background

Quantitative analysis of retinal nerve fibers, often by assessment of retinal nerve fiber layer (RNFL) thickness, is of great significance for the diagnosis and long-term monitoring of optic nerve diseases, such as glaucoma [1, 2]. RNFL thickness is known to be affected by age, axial length or refractive error, optic disc size and content of non-neuronal components [3–9]. Measurements are susceptible to non-negligible errors arising from ocular magnification resulting from axial elongation [10–14] and adjustment for ocular magnification has been found to invalidate the relationship between RNFL thickness and axial length or refractive error [5, 10, 13, 14]. Any relationship between RNFL thickness and optic disc area remains controversial. Indeed, peripapillary RNFL cross-sectional area may give a more accurate quantitative assessment of retinal nerve fibers without the influence of ocular magnification [3, 15] but there have been no previous reports of the peripapillary RNFL cross-sectional area and other parameters. Measurements were made of the peripapillary RNFL cross-sectional area using spectral domain optical coherence tomography (OCT) in a Chinese population during the current work. Correlations between RNFL cross-sectional area and other systemic and ocular parameters were evaluated.

## Methods

### Subjects

Participants were recruited from the Beijing Eye Study 2011, a population-based cross-sectional study including subjects over 50 years of age [16, 17]. Ethical approval was granted by the Medical Ethics Committee of Beijing Tongren Hospital, the study was conducted in accordance with the Declaration of Helsinki and informed consent was given by all subjects.

Exclusion criteria included the presence of diseases such as glaucoma, optic neuropathy, history of intraocular surgery, macular membrane or vitreoretinal traction. Those with blurred color fundus images or OCT images that could not be evaluated were also excluded.

### Data collation

All participants underwent detailed ophthalmic examinations and completed questionnaires [18, 19], as described previously [16, 17, 20]. Ophthalmic examination included 45° color fundus photographs centered on the macula and optic disc (Type CR6-45NM, Canon Inc., USA) and spectral domain OCT (Spectralis OCT; Heidelberg Engineering, Heidelberg, Germany) which were performed after pupil dilation.

A planimetric software program was used to measure the optic disc area from color fundus photographs centered on the optic disc [20]. Ocular magnification was corrected according to Littmann’s method, taking into account the refractive error [20].

**Fig. 1:**
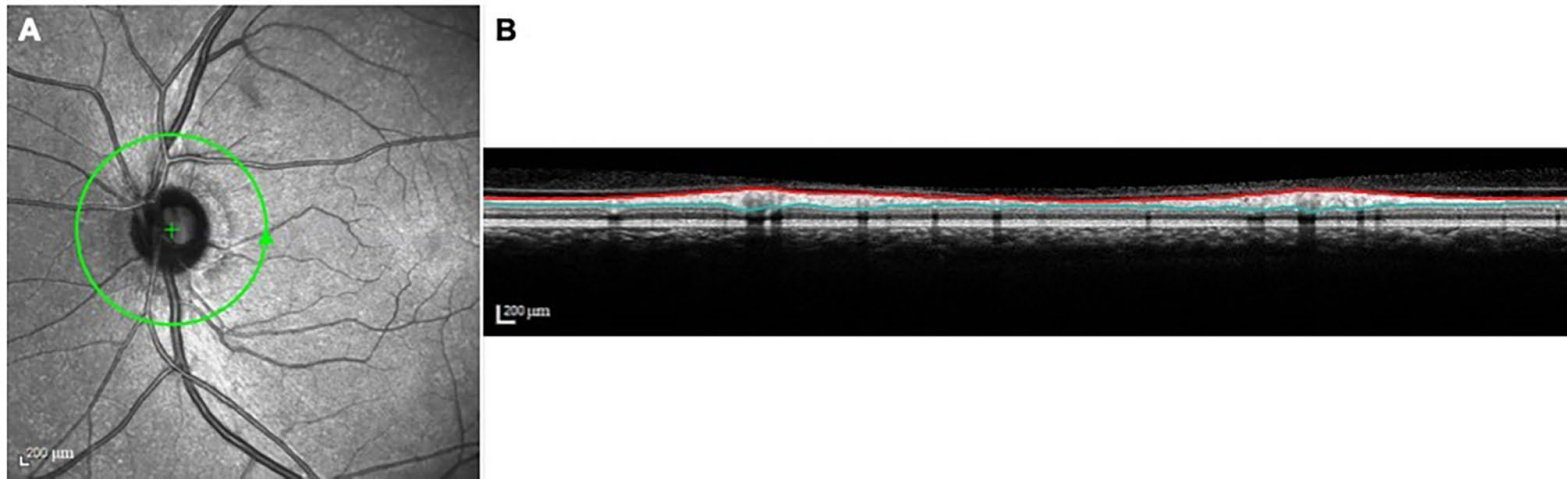
Method of peripapillary RNFL cross-sectional area measurement. Figure **A**: green line indicates peripapillary circular scanning path and arrow indicates a anticlockwise scanning direction for the left eye. The scanning circle diameter was automatically calculated by Heidelberg Eye Explorer software. Figure **B**: cross-section of the retina under the scanning path of Figure A. The red line represents the internal limiting membrane and the green line represents the interface between the retinal nerve fiber layer and the ganglion cell layer. The area between them is the cross-sectional area of the nerve fiber layer

Spectral-domain OCT scans including a peripapillary circular B-scan centered on the optic disc with a diameter of 12°were performed and the peripapillary RNFL cross-sectional area of the right eye was measured. Inner and outer RNFL boundaries were outlined automatically by Heidelberg Explorer (HEE, version 5.3; Heidelberg Engineering, Heidelberg, Germany) and RNFL was re-adjusted manually by a trained operator in cases where misalignment was apparent. RNFL thickness was measured by HEE at 768 equally-spaced spots around the 360° circle and a mean thickness value was generated. The peripapillary scanning circle diameter was calculated automatically by HEE and used to calculate the circumference of the scanning circle. RNFL cross-sectional area was calculated by multiplying the circumference by the mean peripapillary RNFL thickness (Fig. 1).

In addition, the RNFL cross-sectional area of 195 eyes was measured using Image J software to ensure the consistency of the two methods.

SPSS software (SPSS for Windows, version 26.0, IBM-SPSS, Chicago, IL, USA) was used for statistical analysis. The correlation between RNFL cross-sectional area and other parameters was analyzed using univariate linear regression, followed by multivariate linear regression. Standardized regression coefficients beta, regression coefficients B and 95% confidence interval (CI) were generated and the intraclass correlation coefficient (ICC) was calculated for consistency. An ICC > 0.80 was considered to indicate almost perfect reliability. All *p* values were 2-sided and a *p* value < 0.05 was considered statistically significant.

## Results

Peripapillary RNFL cross-sectional area was successfully measured in 2404 subjects with a mean age of 62.2 ± 9.0y (range: 50-93y), mean refractive error of -0.00 ± 1.65 diopters (D; range: -12.00 D to 13.50 D) and mean axial length of 23.14 ± 1.00 mm (range: 19.39–30.88 mm). The cohort included 1054 males (43.8%) and 1350 females (56.2%). The mean peripapillary RNFL cross-sectional area was 1.11 ± 0.12mm^2^ (range: 0.486–1.538 mm^2^) and the mean optic disc area was 2.57 ± 0.48mm^2^ (range: 1.13-5.34mm^2^). The ICC for the 2 methods for measuring RNFL cross-sectional area was 0.912 (*p* < 0.001).

Univariate linear regression analysis showed that larger RNFL cross-sectional area was associated with younger age (*p* < 0.001), female gender (*p* < 0.001), absence of a history of diabetes mellitus (*p* = 0.002), non-smoking (*p* = 0.013), higher level of cognitive function (*p* = 0.013), higher systolic pressure (*p* < 0.001), lower creatinine levels (*p* = 0.006), higher cerebrospinal fluid pressure (*p* < 0.001), higher best-corrected visual acuity (BCVA) (*p* < 0.001), larger optic disc area (*p* < 0.001), lower trans-lamina cribrosa pressure (*p* < 0.001), lower anterior chamber depth (*p* < 0.001) and greater subfoveal choroidal thickness (*p* < 0.001). RNFL cross-sectional area was not associated with height (*p* = 0.431), weight (*p* = 0.904), diastolic pressure (*p* = 0.268), pulse rate (*p* = 0.503), cholesterol level (*p* = 0.068), blood triglycerides (*p* = 0.298), high-density lipoprotein level (*p* = 0.994), low-density lipoprotein level (*p* = 0.103), intraocular pressure (*p* = 0.189), axial length (*p* = 0.868), refractive error (*p* = 0.781), central corneal thickness (*p* = 0.809) or lens thickness (*p* = 0.625). Multivariate analysis showed an association of larger RNFL cross-sectional area with larger optic disc area (*p* < 0.001) after adjustment for younger age (*p* < 0.001), female gender (*p* < 0.001) and history of diabetes mellitus (*p* = 0.002, Table 1).

**Table 1:** Associations of peripapillary RNFL cross-sectional area and other parameters (multivariate analysis)

Parameters	*p*	Standardized coefficient beta	Nonstandardized coefficient B	95%CI of B
Age(y)	< 0.001	-0.214	-0.003	-0.003 to -0.002
Optic disc area	< 0.001	0.201	0.049	0.037–0.061
Gender	0.001	0.080	0.019	0.007–0.030
History of diabetes	0.012	-0.062	-0.023	-0.040 to -0.005

The BMO has been reported to shift backward towards the fovea or to become enlarged in moderate or severe myopia, leading to a reduction or an increase of the ophthalmoscopically visible optic disc area [21–24]. To avoid the error of optic disc area measurement linear regression analysis was repeated after removing 155 subjects with moderate or high myopia (spherical equivalent ≥ -3.00D). Multivariate analysis indicated that the correlation between RNFL cross-sectional area and optic disc area (*p* < 0.001) remained significant after adjustment for age (*p* < 0.001), female gender (*p* = 0.003) and history of diabetes (*p* = 0.030, Table 2).

**Table 2:** Associations of peripapillary RNFL cross-sectional area and other parameters (multivariate analysis, subjects with moderate and high myopia were removed)

Parameters	*p*	Standardized coefficient beta	Nonstandardized coefficient B	95%CI of B
Age(y)	< 0.001	-0.223	-0.003	-0.004 to -0.002
Optic disc area	< 0.001	0.205	0.050	0.038–0.061
Gender	0.003	0.075	0.017	0.006–0.029
History of diabetes	0.030	-0.054	-0.020	-0.038 to -0.002

## Discussion

Spectral-domain OCT was used in the present cross-sectional study of a Chinese population to measure peripapillary RNFL cross-sectional area to enable analysis of relationships with other parameters. A larger RNFL cross-sectional area was significantly associated with younger age, female gender, absence of a history of diabetes mellitus and larger optic disc area.

No correlation was found between peripapillary RNFL cross-sectional area and axial length or refractive error. Previous studies have determined the RNFL to become thinner with increasing axial length [9, 10, 25, 26] but this relationship is thought to be due to ocular magnification. Adjustment for ocular magnification has been found to remove the correlation of RNFL thickness with axial length in numerous recent studies [9, 10, 14, 25, 26], consistent with the findings of the present study. RNFL thickness measurements after adjustment for ocular magnification allow a more accurate quantitative assessment of retinal nerve fibers. We have previously shown that peripapillary RNFL cross-sectional area = ocular magnification adjusted RNFL thickness * consistent scanning circle diameter(usually 3.4 mm) *π and peripapillary RNFL cross-sectional area and ocular magnification adjusted RNFL thickness have very similar significance for quantitative evaluation of RNFL [11, 27].

A positive correlation between peripapillary RNFL cross-sectional area and optic disc area was found during the current work. Previous evaluations of the RNFL and its correlation with optic disc area have proved controversial.

Quigley et al. [28] compared optic disc area in fundus photographs with the number of retrobulbar, myelinated optic nerve fibers estimated from light microscopy in 25 normal monkey eyes and found a linear positive correlation. Jonas et al. [29]. also found a positive correlation between the numbers of optic nerve fibers and optic disc area by using a computer image analyzer to assess histological sections of 72 optic nerves from 56 cornea donors. Other studies have also found a significant positive correlation between RNFL thickness and optic disc area in normal people and glaucoma patients after adjustment for race, gender, age, axial length and other factors [4, 30–32]. Yücel et al. [33]. measured optic disc area in vivo by confocal scanning laser ophthalmoscope with the assessment of optic nerve fiber numbers in histological sections from 10 monkeys with laser-induced glaucoma. Optic disc size was again shown to correlate with the number of optic nerve fibers. In addition, the optic rim area has also been shown to increase with enlargement of the optic disc area [30, 34], indicating the presence of more optic nerve fibers within a larger optic disc area.

However, Balazsi et al. [35]. found no significant correlation between the number of retrobulbar optic nerve fibers and optic disc area during histopathological examination of 16 human eyeballs. Similarly, Mansoori et al. [36]. found no correlation between RNFL thickness and optic disc area when Spectral-domain OCT was used to evaluate the correlation between peripapillary RNFL thickness and optic nerve head parameters in 65 healthy subjects. Other studies have also failed to find a correlation between RNFL thickness and optic disc area [7, 37].

Sample sizes, subject ages and methodological differences in disc size and RNFL measurements (histopathological measurement, confocal scanning laser ophthalmoscope, OCT, color fundus photographs) may explain some of the contradictory findings. However, another influential factor is ocular magnification. The impact of ocular magnification on RNFL thickness measurements has been the subject of frequent previous discussion [9, 10, 25, 26] and optic disc area measurements are also affected by ocular magnification. However, ocular magnification was not corrected in the studies cited above.

David Huang et al. [11]. studied the correlation between RNFL thickness and optic disc area in 196 eyes from 101 normal subjects with refractive error between + 3.0D and − 7.0D. Optic disc area was measured by confocal scanning laser ophthalmoscope and RNFL thickness was measured using OCT. RNFL thickness was shown to correlate positively with optic disc area prior to correction for ocular magnification but the correlation disappeared post-correction.

RNFL cross-sectional area was measured during the current study, rather than ocular magnification-corrected RNFL thickness. We have previously shown that RNFL cross-sectional area and ocular magnification adjusted RNFL thickness have a similar significance for the quantitative evaluation of RNFL [27]. The optic disc area was measured from color fundus photographs and ocular magnification was corrected according to Littmann’s method [26]. By contrast with the results of David Huang et al., we found peripapillary RNFL cross-sectional area to increase with the increase in optic disc area.

The inconsistency between the current study and that of David Huang may be explained, as follows. First, there was a difference in sample size. Huang studied 196 eyes from 101 normal subjects whereas 2404 eyes from 2404 participants were included in the present work. Second, different methods for the measurement of optic disc area were used. Huang used a confocal scanning laser ophthalmoscope whereas measurements were made from fundus photographs centered on the optic disc by a planimetric software program during the current work. Estimation of actual size optic disc area with Littmann’s mathematical approximations from color fundus images has < 4% bias [38, 39] compared with a bias within 2% when disc area is measured using confocal scanning laser ophthalmoscope [40]. A good correlation is shown between these two methods despite some differences in optic disc area measurements [41]. In addition, simultaneous measurements of optic disc area by color fundus photographs and confocal scanning laser ophthalmoscope were made in 52 randomly selected eyes during the present study and no significant differences were found between the two methods [20].

We acknowledge some limitations to the present study. Changes to the ophthalmoscopically visible disc area and shape occur in color fundus images due to the change of BMO caused by the elongation of the eye, resulting in errors in disc area measurements [21–24]. In order to avoid such errors, analysis was repeated after the removal of moderately and highly myopic eyes (spherical equivalent ≤-3.00D) and a positive correlation between peripapillary RNFL cross-sectional area and optic disc area was found. In addition, only subjects over 50 years old with a narrow age range were included and numbers of retinal nerve fibers are known to decrease with age. Further studies of large sample sizes encompassing a greater age range are required to confirm the current findings.

## Conclusions

In conclusion, the current cross-sectional study found a positive correlation between peripapillary RNFL cross-sectional area and optic disc area after correction for ocular magnification, suggesting that eyes with larger optic discs have thicker RNFL. RNFL cross-sectional area is only an estimate of the number of retinal nerve fibers, as is corrected RNFL thickness. No anatomical evidence has been reported to enable correlation of nerve fiber layer thickness in vivo with postmortem retinal nerve fiber number. As a result, it remains unclear whether the relationship between RNFL cross-sectional area and optic disc area is really due to differences in numbers of nerve fibers or other factors, such as magnification or content of non-neuronal components. Further studies with diverse measurement methods and a wider range of subject ages are required to confirm the findings.

## Data Availability

The datasets generated and/or analysed during the current study are not publicly available due to the possibility of invasions personal privacy but are available from the corresponding author on reasonable request.

## Abbreviations

BCVA: best-corrected visual acuity
BMO: Bruch’s membrane opening
CI: confidence interval
D: diopters
HEE: Heidelberg Explorer
ICC: intraclass correlation coefficient
OCT: optical coherence tomography
RNFL: retinal nerve fiber layer
